# Effects of an Intervention for Promoting Basic Motor Competencies in Middle Childhood

**DOI:** 10.3390/ijerph18147343

**Published:** 2021-07-09

**Authors:** Anne Strotmeyer, Miriam Kehne, Christian Herrmann

**Affiliations:** 1 Department of Exercise and Health, Paderborn University, 33098 Paderborn, Germany; miriam.kehne@upb.de; 2 Physical Education Research Group, Zurich University of Teacher Education, 8090 Zurich, Switzerland; christian.herrmann@phzh.ch

**Keywords:** middle childhood, children, motor skills, school sports, sport, training, primary school

## Abstract

The development of motor competencies is necessary for participation in the culture of sport, exercise, and physical activity, which in turn supports the development of a healthy lifestyle. A lack of physical activity in childhood and deficits in motor performance emphasize the relevance of interventions for promoting basic motor competencies. However, there are research desiderata with regard to such interventions. This article describes an intervention program for promoting basic motor competencies in middle childhood (around 6 to 10 years of age). The intervention was investigated in a longitudinal study from June 2019 to January 2020 (*n* = 200; 58% girls, *M* = 8.84 years, *SD* = 0.63) at three primary schools. The intervention was conducted once a week in physical education (PE). The comparison group participated in regular PE. The intervention showed significant effects on basic motor competencies in *object movement* but not in *self-movement*. The results demonstrate that positive effects on basic motor competencies can be achieved with the help of a relatively simple intervention. Further longitudinal studies are desirable as a means of substantiating the results and developing evidence-based concepts to support children in their development in the best possible way.

## 1. Introduction

With regard to physically active behavior, children need to develop basic motor competencies to lead a healthy lifestyle and participate in the culture of sport and exercise [1,2]. The promotion of basic motor competencies in middle childhood (around 6 to 10 years of age) is becoming increasingly important for several reasons. First, the level of motor performance in middle childhood declined almost 6% from 1965 to 2005 in Germany and has since stagnated (e.g., [3,4]). Deficits in motor performance in middle childhood are also evident from an international perspective. This is shown in the systematic review “Global levels of fundamental motor skills in children” by Bolger and colleagues [5]. The review includes 64 articles from 2004 to 2019. The results show that preschool-aged children (3–5 years) worldwide demonstrate average levels of motor skills, while children aged 6–10 years demonstrate below-average levels [5]. Moreover, studies show that around a quarter of children need support on whole-body coordination (*self-movement*: 24%, boys: 26%; girls: 21%) and on ball control (*object movement*: 25%, boys: 13%; girls: 39%) [6]. Furthermore, girls tend to achieve poorer performances on *object movement* and boys on *self-movement* than their opposite-sex peers [6] (cf. [7]). Second, it is critical with regard to the assumption that motor deficits are responsible for the lack of physical activity [2,8] that the WHO recommendation of 60 min per day for health-related physical activity [9] is met by just a third of three- to seventeen-year-olds (girls: 22.4%, boys: 29.4%; [10,11]). This is important because studies guided by the conceptual model by Stodden and colleagues [12] show that physical activity levels are related to motor competence levels and also to the accuracy of perceived motor competence [2,13,14,15]. Third, the increasing shift of children’s living environment to school is leading to an increase in sedentary behaviors [16,17,18,19,20]. Programs for promoting movement, games, and sports are therefore gaining importance in all-day schools [17,21]. In addition, the development of motor competencies, which form the basis for participation in health-oriented physical activity across the lifespan, requires structured programs (cf. [22]). However, there is a general lack of research on the promotion of motor competencies in the field of sports pedagogy [23,24,25]. There are only a few intervention studies in the primary school setting that aim explicitly at promoting motor competencies. In general, the studies show positive effects on object control (ball skills), locomotion (gymnastics), or at least one motor skill (e.g., [22,26,27,28,29]). Most of the interventions were conducted by means of additional programs. Existing meta-analyses reveal the inconsistency of methodological approaches across the studies in terms of intervention programs and measuring instruments [26,27]. For example, the intervention programs differed with regard to the age range of the target group (3–10 years), the length of the intervention (6–15 weeks; 480–1140 min), the implementation (e.g., by parents, scientists, teachers), and the content [27]. No relationship between length of intervention and effect size could be ascertained [27]. Furthermore, programs commonly provide neither the theoretical foundation nor specific information focusing instead on intervention effects (cf. [22,27]). Thus, Logan and colleagues recommend implementing structured motor promotion programs to support motor development in childhood and highlight the need for future research on the feasibility of the structured promotion of motor performance [27]. In addition, the term motor competence (or competency) is usually used as a general term for various motor performance dimensions (e.g., motor ability, motor coordination motor proficiency, motor performance, and fundamental movement/motor skill or FMS for short) to describe goal-directed human movement (cf. [2]). Accordingly, there are great differences between the findings of interventions on outcome parameters, such as coordination ability (e.g., [30]), motor performance (e.g., [31]), and FMS (e.g., [32]). Thus, the results of the studies do not allow reliable comparisons.

The construct of basic motor competencies is used in sports pedagogy in the German-speaking world. Basic motor competencies are understood as performance dispositions for situation-specific demands (for definition see 2.1) [33]. Currently, this young line of research is dedicated to diagnosis and effects in school sports. Two areas of competence have been defined so far: *object movement* and *self-movement*. Further areas of competence are under development [33]. However, there are questions concerning the design of interventions that specifically addresses basic motor competencies. To our knowledge, to date there have been no studies conducted focusing on the structured promotion of basic motor competencies (by definition).

Against this background, we developed a theoretically derived intervention for the promotion of basic motor competencies in middle childhood [34]. This article refers to the presentation of an intervention study conducted in a primary school under the conditions of a field study. Moreover, in this article we present our analysis of the present findings with regard to the body of research on basic motor competencies and with regard to the stability of motor development.

In the following sections, we briefly describe the concept of the intervention. Then we present the research methodology and the results.

## 2. Intervention Concept

The intervention is intended as a competency-based concept which aims to create a foundation for participation in the culture of sport and exercise. This is also the objective of primary school sports (e.g., [35,36,37]). Promotion of the ability to move, knowledge on movement, and motivation for movement play a central role in achieving this objective [38]. In the view of school compatibility (e.g., [35]), the concept takes the framework for school sports, such as heterogeneity and limited movement time associated with school sports, into account (cf. [39,40]). On the basis of the (sports) pedagogical understanding of competence [38] and the associated importance of capacity for action in sports [41], the methodological-didactic organization of the intervention concept comprises the goal components: (1) *movement ability*; (2) *movement knowledge*; and (3) *movement motivation*. On account of limited movement times, as well as the physicality of the subject of sport, the intervention focuses on promoting the movement ability and knowledge, although the motivation to move also plays a role (for details, see [34]). It should be noted that the content of the concept is available as a manual.

We begin by explaining the term basic motor competencies, because it forms the main foundation for the intervention concept. In the following, we explain the methodological-didactic design, the content, and the scope of the intervention.

### 2.1. Definition of Basic Motor Competencies

The construct of basic motor competencies can be anchored in a pedagogical and a curricular context. It is based on the concept of competence [38] and on the question of what a child should be able to perform at a certain grade level in order to be able to participate in PE and in the culture of sport and exercise [42]. Two competence dimensions were determined: *self-movement* and *object movement*. *Self-movement* focuses on whole-body coordination, and *object movement* aims at ball control. Basic motor competencies are defined as follows by Herrmann, Gerlach, and Seelig [43]:

“Basic motor competencies may be understood as performance dispositions that develop from the demands of specific situations. They help students to meet concrete demands in the culture of sport and exercise, and:

- Can be learned and retained in the long term and take into account previous experiences;
- Are explicitly context-dependent and refer to situation-specific demands in the culture of sport and exercise;
- Are functional performance dispositions that manifest themselves in behavior that is oriented toward mastery.”

[44] (p. 110)

In addition, there are technical motor abilities that are relevant across all sports (endurance, strength, coordination, speed, and agility). They may be equated with physical performance or fitness and can be trained but not learned. The sport-specific motor skills (e.g., throwing technique) focus on the technique and the quality with which the movement is executed. They can be learned and are categorized into open and closed skills according to their degree of variability [33,45].

**Figure 1:**
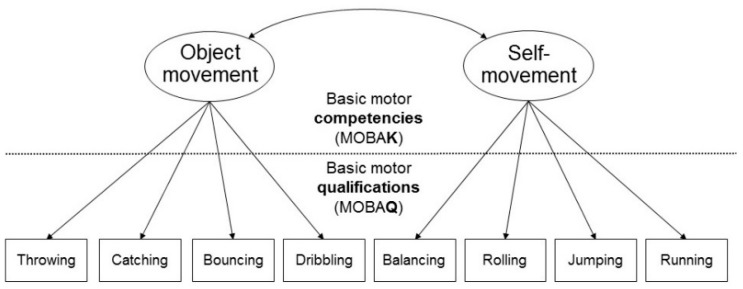
Competency structure model of basic motor competencies for the third grade [47].

Basic motor competencies are demonstrated in the mastery of different demands (e.g., throwing a ball at a target) requiring a combination of motor abilities and skills. They also include a cognitive component (does the student know how strongly he has to throw the ball?) and a motivational-volitional component (does the student make an effort?) [43]. Since it is the interplay of these aspects that determines how well the task is performed, basic motor competencies are considered a partial prerequisite for participation in the culture of sport and exercise [43,46]. Basic motor competencies are not directly observable and are thus differentiated from basic motor qualifications, which can also be formulated as can-do statements (e.g., “can throw,” “can catch”) (cf. Figure 1) [43] (p. 61).

### 2.2. Methodological-Didactic Design of the Intervention

The methodological-didactic organization of the intervention is based on the three goal components: (1) *movement ability*; (2) *movement knowledge*; and (3) *movement motivation*. The focus of the intervention is on motor development. Problem-oriented movement tasks and movement games thus play a key role in the implementation of the intervention.

With regard to (1) *movement ability*, there are two important aspects of the concept. The first is an age-appropriate, varied implementation in which the children can practice the movement patterns in movement games. The second is methodological principles of coordination training via the modification of information demands (optical, acoustic, tactile, kinesthetic, vestibular) and pressure conditions (precision, time, complexity, situation, strain from pressure) of movement patterns to create different coordinative levels of difficulty [48]. The intervention provides for a greater focus on pressure conditions, which is determined by promoting the movement patterns involved in movement games. The intervention program allows for variations in the following aspects: movement goal (functional change), movement feature (speed), movement structure (omission or addition of movements), material and environmental conditions (surface/obstacles), sphere of action (field size, individual/partner work), and environment (cooperating partners).

With the aim of imparting (2) *movement knowledge*, the intervention includes thematic introductions on the significance of learning and performing the movement patterns. In addition, the demonstration and explanation of the performance of movement patterns serve to build up movement knowledge. This knowledge about central aspects of the performance of movements is to be tested and expanded in the course of the unit. This is achieved through varied movement games that offer specific variations of the movement patterns. As a means of enabling the children to build up knowledge of their own ability, this component includes self-reflection exercises at the end of the unit. These exercises begin with a self-assessment in the form of a “can-do task check” [49]. The children are asked to assess their ability using their thumb so that everyone can see it. They rate their ability to perform the movements with a thumbs-up gesture (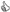) for “good,” a thumbs-middle gesture (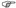) for “could be improved,” and a thumbs-down gesture (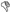) for “not good”. This is followed by an expert panel in which the children give each other tips. By assessing their own abilities and providing a visible expression of this assessment (using their thumbs), the children can see and experience their learning progress and gain awareness of their own motor competencies. In addition, the children-centered expert panel helps them to consolidate the movement knowledge they have acquired.

To promote (3) *movement motivation*, the intervention aims to enable recurring experiences of success. In situations where children are characterized by personal effort, the goal is to praise the children for their effort, as this has a positive reinforcing effect on behavior [50]. However, criticism should be expressed when children do not make an adequate effort even though they meet the performance requirements [51]. According to the self-determination theory of Deci and Ryan [52], autonomy, the feeling of self-determination, is essential for achievement motivation (cf. [53]). Thus, the program is designed to support the children in achieving a realistic self-perception of their motor competencies. With regard to the integration of self-determined learning, the children are encouraged to choose different materials to master the movement patterns and different ways of performing them, as well as trying out new ideas. *Movement motivation* is also supported by movement games in groups and teams. In addition, movement patterns and games are designed specifically to be challenging and motivating as a means of increasing the children’s willingness to make an effort (for details, see [34]). 

### 2.3. Content and Scope of the Intervention

The core of the intervention is formed by elementary movement patterns that are typically cultivated in the course of middle childhood motor development [54,55,56,57]. These patterns can be assigned to the competency domains *self-movement* and *object movement*.

With regard to the framework of school sports and the possibility of implementation in PE classes, the intervention includes 16 units of 45 min (active time: 35 min) (see Table 1). 

Each unit starts with time for free play. Afterwards, the children are welcomed and receive an explanation and demonstration of the movement patterns addressed in the unit (5 min). For a warm-up (5 to 8 min), a movement game on the topic of the unit is included. The main part (15 to 20 min) contains movement tasks and games which are designed specifically to be challenging and motivating. Here, the children have to choose the level of coordinative difficulty regarding the performance of the movement task. Each unit ends with a cool-down, self-reflection exercises, and the children-centered expert panel (5 to 8 min).

In the following, movement tasks and games from the main parts of the first and second units on throwing and catching respectively (hereinafter Unit I, II) are presented in terms of intensity, repetitions, comfort, and difficulty. It should be mentioned that no age-specific or gender-specific differentiations are provided, but rather different coordinative levels of difficulty. In addition, gender-stereotypical content (such as soccer or dance) is not addressed.

After the ten-minute introduction to throwing and catching, the children solve throwing tasks in the main part of Unit I. The exercise consists of ten throwing tasks, which are to be completed one after the other. The difficulty increases with each task, while the number of repetitions decreases. Task one contains ten repetitions, task ten only one repetition. The tasks should be performed in pairs so that the children can give each other feedback. The children perform the throwing tasks for 15 min. In Unit II, coordinative aspects are first addressed with the help of balance demands. The children balance sideways over the benches one after the other and try to throw the ball from the bench to the wall and catch it again without falling off the bench. The pressure of complexity (achieved by turning the benches around) and the pressure of strain (achieved by placing the benches at an angle to the wall) is gradually increased. This exercise takes place for ten min. Afterwards, the focus is on playful practicing. For this purpose, the playing field is divided into two halves. In the middle of the two fields there are two small boxes, each with a marker hat on it. Outside the playing field, a bench is placed for each team to serve as a “waiting place”. The goal is to throw off the children in the opponent’s field and thus send them to the bench. There are two ways to get back into your own field. In the first, if the ball is successfully intercepted by the opponent, the foremost child on the bench may return to the field. In the second, if a teammate hits one of the hats on the box so that it falls to the ground, all teammates on the bench are free again. The further the small boxes are placed from the center line, the higher is the pressure of precision. This is adjusted in terms of the children’s ability level. Ten min are scheduled for the game.

The content design of all units is summarized in a manual that allows a standardized implementation of the intervention program. 

### 2.4. Formative Evaluation and Feasibility of the Intervention 

We evaluated the development of the intervention concept parallel to the process. This allowed us to also consider the practical feasibility of the intervention in addition to its theoretical and empirical aspects. In order to test the practical feasibility in a realistic way, both the testing coaches and the tested children were specifically interviewed during the intervention process. A central result of the formative evaluation is that the intervention is feasible and practical with heterogeneous groups. In addition, the children’s evaluation of the program implementation shows that the intervention is rated just as positively as regular PE lessons. The intervention appealed particularly to children with weak and more average basic motor competencies, and to girls. Overall, the most striking result of the intervention concerned the children’s subjective assessment of their learning progress. Whereas the children of the intervention group emphasized their learning success, the children of the control group did not acknowledge any learning progress. The objective test results confirmed the children’s impression (see below).

The central aim of this study was to determine whether the presented intervention is effective in improving basic motor competencies in middle childhood when compared to a control group. Another goal was to investigate whether the results of the present study fit into the body of research on basic motor competencies and whether children’s performance shows stability in the development of basic motor competencies. 

## 3. Materials and Methods

### 3.1. Design and Procedures

The study was conducted from June 2019 to January 2020 at three primary schools in North Rhine-Westphalia (Germany) and included two measurement points (t1: June 2019; t2: January 2020). In accordance with the field study design and for organizational reasons, the intervention was conducted in PE classes. Schools had similar conditions with regard to the number of students, school district (all schools were located in the same district), space (in a gymnasium), teacher resource, and timetable. The study ran for a total of eight months (not including summer and fall break). 

The study was conducted according to the guidelines of the Declaration of Helsinki. After the study was approved by the University of Paderborn Ethics Committee (date of approval 15 May 2019), the principals of the participating schools gave their approval for the study to be carried out. Information on participation in the study and on its procedures was provided in letters sent by the school principals to the parents or legal guardians of the participating children. Data were collected on children whose parents or legal guardians submitted written declarations of consent.

The children were cluster randomized at the class level into an intervention group (IG) and a comparison group (CG). In the first half of the 2019/2020 school year, the IG received one standardized 45 min intervention unit each week in place of a regular PE lesson. The CG received regular PE lessons. The units were interrupted for class field trips and fall break. In four of the six intervention classes, it was possible to complete 100% of the intervention. In the other two intervention classes, only 13 of 16 units could be conducted for organizational reasons on the part of the school, and these classes thus completed only 81.3% of the intervention.

The intervention was conducted by the research group leader and two qualified PE students over the whole intervention period of six months. The teachers were present in the gymnasium while the intervention was being conducted but did not take part in the intervention. 

Following the completion of each unit, the intervention leaders documented the content and course of the unit, the children’s motivation, and any special incidents in order to ensure a standardized implementation and assess fidelity during the intervention. The documentation indicates that a standardized implementation of the intervention was successful. 

### 3.2. Sample

The sample included a total of 200 children (84 boys, 116 girls; age: *M* = 8.84 years, *SD* = 0.63, age range: 7.75–10.42 years; BMI: = 16.48, *SD* = 2.46) from six fourth grade and five third grade classes from three primary schools in North Rhine-Westphalia. The description of the sample for the IG and the CG is provided in Table 2.

### 3.3. Measures

We measured the children’s basic motor competencies with the MOBAK 3–4 test (in German: Motorische Basiskompetenzen; MOBAK for short) [58,59]. MOBAK 3–4 is divided into the two competency domains *self-movement* and *object movement*. *Self-movement* includes the test items balancing, rolling on a box, rope jumping, and running. *Object movement* contains the test items throwing at a target, throwing and catching, bouncing, and dribbling (cf. Figure 1). 

The reliability and validity of the MOBAK 3–4 test were demonstrated in the validation study conducted previously using confirmatory factor analyses (CFI = 0.97; RMSEA = 0.033) [60]. The results are consistent with the MOBAK 1–4 Test Manual [58].

The MOBAK 3–4 test was carried out in station operation in the school gymnasium. Each station consisted of two test items. The measurements were carried out by twelve testers trained in the application of the MOBAK 3–4 test. Eight of the testers were responsible for the measurement (one task per tester), the rest for the organization. The children performed the eight tasks in a group of four to six children. At each station the tester explained how the motor task is performed and then demonstrated it. In accordance with the description of the instrument, each child performed two attempts (except for the throwing and catching tasks, where they had six attempts), without previous trial attempts. The approximate duration of the application of all MOBAK 3–4 items was 45 to 60 min, depending on class size.

Age and sex were documented, and body weight and height were measured by the testers.

### 3.4. Statistical Analyses

Theory-based sample size estimation was conducted using G * Power [61]. According to this analysis, a sample size of *n* = 172 was necessary for a significant moderate effect (Power = 90%; *f* = 0.25) [62]. 

The data were processed and frequency and correlation analyses conducted with SPSS 26 [63]. The children’s body weight and height were used to calculate their body mass index (BMI = weight [kg]/height^2^ [m^2^]). 

For the descriptive analysis, differences between the IG and the CG were tested specifically for motor competencies, sex, BMI, and age by means of the Mann-Whitney U test. Furthermore, mean values, standard deviations, and 95% confidence intervals in *self-movement* and *object movement* were calculated at the first and second measurement points for the IG and the CG as well as for the girls and the boys. 

To analyze gender-specific differences in the children’s level of motor competency, we conducted *t*-tests.

Correlations between *self-movement* and *object movement* as well as with age, sex, (m = 1; w = 2), and BMI were calculated with the help of Pearson’s correlation analysis, allowing analysis of the results with regard to the body of research on basic motor competencies and the stability of motor performance. 

For the statistical inference analysis, we conducted analyses of variance with repeated measurements with the factors: (1) time (*self-movement* and *object movement* at t1 and t2); (2) time × group (IG and CG); (3) time × sex; and, in order to represent differential gender-specific effects, (4) time × group × sex. 

For the MOBAK 3–4 test items, 10 values were missing at the first measurement point and 20 were missing at the second measurement point. For sex, we were able to include the complete data of all children. For the BMI, there were 11 missing values. Due to the low number of missing values, we excluded the children with missing values in applying these variables. The basis for the analysis of variance is data sets with related data for the first and second measurement point (*n* = 170). 

The significance level was set at *p* < 0.05. To determine the strength of the effect (partial *η^2^*), we calculated the effect size (*f*). The effect sizes were interpreted as small (*f* = 0.10), moderate (*f* = 0.25), and large (*f* = 0.40 large) [52]. 

## 4. Results

### 4.1. Descriptive Statistics

Table 2 provides baseline information (t1) including age, sex, height, weight, and BMI, by IG and CG. While there were no differences between the IG and the CG in the variables BMI (*U* = 4525.00, *Z* = −0.41, *p* = 0.685) and sex (*U* = 4341.00, *Z* = −1.70, *p* = 0.090), the children in the IG had a lower average age (*U* = 6573.50, *Z* = −4.13, *p* < 0.000).

The motor competencies in *self-movement* and *object movement* of all children as well as of the IG and the CG, including gender-specific results, are provided in Table 3. In *object movement*, the boys significantly outperformed the girls at both measurement points (t1: t (188) = 6.10, *p* < 0.000; t2: t (178) = 5.31, *p* < 0.000). In *self-movement*, the girls performed significantly better than the boys only at the first measurement point (t1: t (188) = −3.05, *p* = 0.003); t2: t (178) = −1.61, *p* = 0.110).

At the first measurement point there were differences between the IG and the CG in *object movement* (t (188) = −2.28, *p* = 0.024) but not in *self-movement* (t (188) = −1.33, *p* = 0.188) (cf. Table 3).

### 4.2. Analysis of Correlations between Self-Movement and Object Movement and Sex, Age, BMI

The correlation analyses reveal that the children’s performances remained stable in the competency domains *self-movement* and *object movement* over both measurement points. There were strong correlations between performances at the first and second measurement point in *self-movement* (*r* = 0.60, *p* < 0.001) and in *object movement* (*r* = 0.69, *p* < 0.001). Age was weakly correlated with *object movement* at both measurement points (see Table 4). The older children thus had slightly higher motor competencies in object movement than the younger children. There was no correlation between age and *self-movement.* Sex was moderately correlated with *object movement* at the first measurement point (*r* = 0.42, *p* < 0.001) and at the second measurement point (*r* = 0.37, *p* < 0.001). The boys were superior to the girls in *object movement.* There were weak correlations between sex and *self-movement* only for the first measurement point (*r* = 0.22, *p* < 0.001). The girls showed a higher competency level than the boys in *self-movement*. BMI was weakly to moderately negatively correlated only with *self-movement* at the second measurement point (*r* = 0.25, *p* < 0.001). Thus, BMI was accompanied by a lower motor competency level in *self-movement*. In *object movement*, no correlation with BMI was found (cf. Table 4).

### 4.3. Analysis of Variance with Repeated Measurements

#### 4.3.1. Factor Time

The analysis of variance with repeated measurements showed that the children significantly increased their performances in *self-movement* from the first to the second measurement point (*F* (1, 166) = 50.12; *p* < 0.001; partial *η*^2^ = 0.23, *f* = 0.55). A significant effect may also be seen in *object movement* (*F* (1, 166) = 33.45; *p* < 0.001; partial *η*^2^ = 0.17, *f* = 0.45); the children improved their performances over time in this competency domain as well. 

#### 4.3.2. Factors Time × Group

**Figure 2:**
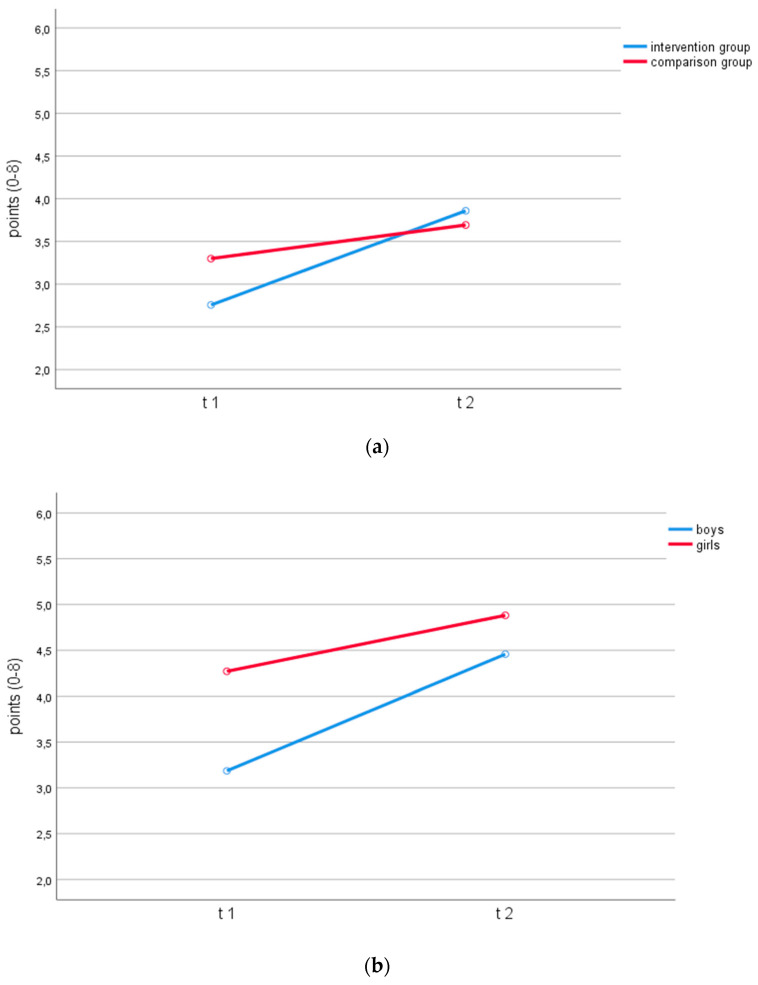
(**a**) Motor competencies of intervention and comparison group in *object movement*. (**b**) motor competencies of boys and girls in *self-movement*.

With regard to the performances in *object movement*, there was a significant interaction between time and group with a moderate effect size (*F* (1, 166) = 7.52; *p* = 0.007; partial *η*^2^ = 0.04, *f* = 0.21). This is an indication of a positive effect of the intervention on basic motor competency in *object movement* (see Figure 2a). In *self-movement*, there was no interaction effect between time and group (*F* (1, 166) = 0.25; *p* = 0.619; partial *η*^2^ = 0.00; *f* = 0.03).

#### 4.3.3. Factors Time × Sex

There was a significant interaction between time and sex in *self-movement* (*F* (1, 166) = 6.12; *p* = 0.014; partial *η*^2^ = 0.04, *f* = 0.19). The girls achieved a higher number of points than the boys at both measurement points. However, the boys improved more than the girls in self-movement (see Figure 2b). In *object movement*, there was no interaction effect between time and sex (*F* (1, 166) = 1.07; *p* = 0.303; partial *η*^2^ = 0.01; *f* = 0.08).

#### 4.3.4. Factors Time × Group × Sex

There were no interaction effects between time, group, and sex in *self-movement* (*F* (1, 166) = 0.67; *p* = 0.413; partial *η*^2^ = 0.00; *f* = 0.06) or in *object movement* (*F* (1, 166) = 0.47; *p* = 0.495; partial *η*^2^ = 0.00; *f* = 0.06). Thus, the gender-specific interaction effect in *self-movement* described above appeared regardless of group membership.

## 5. Discussion

The major aim of the present study was to investigate the effects of a theoretically derived intervention for promoting basic motor competencies in middle childhood. Further objectives were to investigate whether the results of the present study fit into the body of research on basic motor competencies and whether children’s performance shows stability in the development of basic motor competencies.

For study design reasons, the intervention was conducted in PE classes. However, the intervention could also be carried out in extracurricular school sports (e.g., in all-day schools) or in other sports settings (e.g., club sports) for two or three times a week. The intervention took place once a week (active time: 35 min) in the class of the IG over the course of half a school year. The CG received (regular) PE lessons, as usual. The study focused on changes in basic motor competencies resulting from the intervention and on gender-specific differences. 

First, it should be noted that the present cross-sectional findings from MOBAK 3–4 fit seamlessly into the body of research on basic motor competencies in relation to the specific findings regarding sex, age, and BMI [6,60]. To our knowledge, this is the first study to examine longitudinal findings from MOBAK 3–4 with regard to the stability of basic motor competencies. The results show a moderate to high stability of basic motor competencies in *self-movement* and *object movement*. This is similar to findings from a longitudinal study with first and second graders (*n* = 1031, 54 % boys, *M* = 6.83 years, *SD* = 0.44) tested with MOBAK−1 [64]).

To date, no intervention studies on the promotion of basic motor competencies in the sense defined above are known. The body of research on the promotion of motor performance in the primary school setting shows a broad range of intervention studies that differ in design, characteristics, and measurements. Thus, findings of the studies do not allow reliable comparisons.

Nevertheless, in contrast to previous research on the promotion of motor performance in middle childhood [26,27], the present findings show no significant intervention effect in *self-movement* but only a gender-specific effect. It remains a matter of speculation why the intervention obtained no positive effect in *self-movement*. It is possible that the content in *self-movement* was not sufficiently challenging. Although the girls showed a higher motor competency level in *self-movement* in general, the boys showed greater improvements in their motor competencies in *self-movement*. This could be an indication that it is possible to cause positive changes in *self-movement* in boys over a shorter period of time than it is in girls, although the boys had a lower baseline level. This could possibly be explained by the fact that boys tend to participate in sports that require competencies in *object movement* and thus object control [65]. However, the significant intervention effects in *object movement* found in this study are in line with the state of research (e.g., [26,27]). For example, Lee and colleagues showed in an intervention study (*n* = 31; aged = 6.65 ± 0.98) using an 8-week FMS-based afterschool program (addressing 12 basic motor skills) that a structured intervention (e.g., fun games and goal setting) can promote locomotor and object control skills in addition to physical activity in the primary school setting [22]. However, in contrast to the present study, the duration and frequency (three times per week; 60 min each time) were higher in the study by Lee and colleagues. Costello and Warne conducted an intervention study (*n* = 100; aged 9 ± 1) over four weeks addressing one FMS (single leg hop, standing broad jump, the overarm throw, and sprint run) in 30-min sport lessons two times a week [28]. The results show significant improvements in FMS. Also in this study, the frequency of intervention was higher compared to the present study. Furthermore, Lopes and colleagues examined the effect of a ten-month intervention to improve motor skills of primary school children (*n* = 60; aged 9 ± 1) in three classes [29]. In contrast to the present study, Lopes and colleagues assigned classes to three diverse conditions. Class 1 received an intervention in PE lessons two times/week (PE−2), class 2 received the intervention three times/week (PE−3), and class 3 did not have any PE lessons (control group). The intervention contained a specific sport program to promote “skills from gymnastics, soccer, Olympic handball, basketball, and track and field” (p. 3). Both intervention groups (PE−2,3) showed significant improvements in gymnastics and handball. Only PE−3 improved soccer skills (d = 0.55), and only PE−2 improved basketball skills (d = 0.46) [29]. Similar to the intervention programs of Costello and Warne as well as Lopes and colleagues, the present intervention program ran for 45 min (active time: 35 min) with regard to the possibility of implementation in the school context [28,29]. Compared to those studies, however, the frequency of implementation in this study was lower.

In summary, this article describes the development and implementation of the first theoretically derived intervention for promoting basic motor competencies in heterogeneous groups with the focus on movement, games, and sports. The competence-oriented concept is based on the basic requirements for health-oriented physical activity. It includes aspects for the promotion of *movement ability, movement knowledge,* and *movement motivation*. In this context, the promotion of self-assessment is also central. For example, Burns and colleagues show that physical activity and enjoyment of physical activity are strongly dependent on motivational constructs such as perceived competence and self-efficacy [66]. In contrast to studies that focus on motor skills and abilities, and thus on physical performance or fitness and the quality of the movement execution, this study focused on motor competencies, and thus on the situation-specific (successful) accomplishment of movement tasks. The intervention concept was designed to maximize movement time without focusing on monotonous training (e.g., station training). Instead, basic motor competencies were promoted in a playful manner in age-appropriate games. For this purpose, the content was designed to allow the children to select different levels of coordination difficulty. In view of the studies by Lee and colleagues and Gu and colleagues [22,67], the goal-setting strategy appears to be beneficial for further studies on the promotion of motor competencies.

## 6. Limitations

A possible bias in the results could be explained by the study design. This is due to the fact that the study was carried out in the field, and thus had a predetermined framework. First, the children in the IG had a significantly lower mean age. Second, the proportion of girls was 12.6% higher in the IG than in the CG, although this was not significant. In accordance with the arrangement with the primary schools, the intervention was to be implemented once a week in place of one of the planned PE lessons. For organizational reasons on the part of the school, however, intervention units occasionally had to be canceled. In addition, the intervention could not always be conducted continuously every week on account of field trips or project days. With an increase in the frequency and the duration of the intervention, and a continuous implementation, the intervention may be expected to lead to higher positive changes in motor competency levels. Moreover, we would like to note that the intervention study was originally planned as a crossover study with three measurement points over a duration of 12 months (June 2019 to June 2020). Due to the coronavirus pandemic and the temporary school closures, however, we had to modify the intervention by dropping the third measurement point as well as the exchange of the intervention and comparison group. 

## 7. Conclusions

The results of the study demonstrate that it is possible to achieve positive effects on basic motor competencies with the help of this low-threshold and relatively short competency-based intervention. It is remarkable that the intervention was conducted not in addition to, but rather in the context of, PE classes. Due to the small scope of the intervention (16 units of 35-min active time each) as well as the low implementation frequency (1 ×/week), we assume that increasing the scope and frequency of the intervention would lead to an even greater increase in basic motor competencies.

Further longitudinal studies would be desirable, not only as a means of validating the results, but also with a view to developing evidence-based recommendations for promoting basic motor competencies to provide children with the best possible support in their development. In light of recent studies on the relationship between actual and perceived motor competencies [7,13,14,15,68], we also recommend including self-perception in future studies.

## Figures and Tables

**Table 1:** Scope and movement patterns of the intervention.

Competency Domains	Movement Patterns	Units
self-movement	running & jumping	3
	rolling & balancing	4
object movement	throwing & catching	3
	bouncing & dribbling	4
repetition	running, jumping, rolling, balancing, throwing, catching, bouncing, & dribbling	2

**Table 2:** Baseline information of intervention (IG) and comparison group (CG).

Variables		IG	CG
*n*	total	114	86
	3rd grade	78	24
	4th grade	36	62
Sex	boys	36.8%	49.4%
	girls	63.2%	50.6%
Age	years (*M ± SD*)	8.68 ± 0.62	9.05 ± 0.59
Height	cm (*M ± SD*)	135.70 ± 6.62	139.97 ± 7.83
Weight	kg (*M ± SD*)	30.55 ± 6.16	32.77 ± 6.99
BMI	kg/m^2^ (*M ± SD*)	16.40 ± 2.50	16.59 ± 2.42

**Table 3:** Descriptive statistics in *self-movement* and *object movement* of IG and CG plus girls and boys.

Groups			Self- Movement Total	Object Movement Total	Self- Movement Girls	Self- Movement Boys	Object Movement Girls	Object Movement Boys
t1	IG	*M ± SD*	3.68 ± 1.95	2.46 ± 2.21	3.95 ± 1.99	3.20 ± 1.81	1.66 ± 1.66	3.86 ± 2.35
		*95% CI*	[3.28–4.07]	[2.01–2.91]	[3.44–4.46]	[2.58–3.82]	[1.23–2.08]	[3.05–4.67]
	CG	*M ± SD*	3.92 ± 2.02	3.26 ± 2.11	4.59 ± 2.07	3.17 ± 1.69	2.51 ± 1.89	4.09 ± 2.06
		*95% CI*	[3.45–4.39]	[2.77–3.75]	[3.92–5.26]	[2.59–3.75]	[1.90–3.13]	[3.38–4.79]
	total	*M ± SD*	3.78 ± 1.98	2.81 ± 2.20	4.20 ± 2.04	3.19 ± 1.74	1.99 ± 1.80	3.97 ± 0.22
		*95% CI*	[3.48–4.08]	[2.47–3.14]	[3.80–4.60]	[2.77–3.60]	[1.96–2.35]	[3.45–4.50]
t2	IG	*M ± SD*	4.63 ± 1.85	3.57 ± 2.11	4.74 ± 2.05	4.43 ± 1.46	2.80 ± 1.66	4.91 ± 2.16
		*95% CI*	[4.25–5.00]	[3.15–4.00]	[4.21–5.26]	[3.93–4.93]	[2.38–3.23]	[4.17–5.66]
	CG	*M ± SD*	4.77 ± 1.74	3.66 ± 1.97	5.03 ± 1.86	4.49 ± 1.58	3.13 ± 2.02	4.26 ± 1.76
		*95% CI*	[4.37–5.17]	[3.21–4.12]	[4.42–5.63]	[3.94–5.03]	[2.47–3.78]	[3.65–4.86]
	total	*M ± SD*	4.69 ± 1.80	3.61 ± 2.04	4.85 ± 1.97	4.46 ± 1.51	2.93 ± 1.81	4.59 ± 1.98
		*95% CI*	[4.42–4.96]	[3.30–3.92]	[4.46–5.24]	[4.10–4.82]	[2.57–3.29]	[4.11–5.06]
Δ t2-t1	IG	*M ± SD*	0.95 ± 1.60	1.12 ± 1.60	0.79 ± 1.57	1.23 ± 1.77	1.15 ± 1.52	1.06 ± 1.77
		*95% CI*	[0.61–1.82]	[0.79–1.44]	[0.38–1.19]	[0.62–1.84]	[0.76–1.54]	[0.45–1.66]
	CG	*M ± SD*	0.85 ± 0.18	0.41 ± 0.17	0.44 ± 1.83	1.31 ± 1.64	0.62 ± 1.59	0.17 ± 1.79
		*95% CI*	[0.44–1.27]	[0.01–0.80]	[−0.16–1.03]	[0.75–1.88]	[0.10–1.13]	[−0.44–0.79]
	total	*M ± SD*	0.91 ± 1.71	0.81 ± 1.67	0.65 ± 1.68	1.27 ± 1.69	0.94 ± 1.56	0.61 ± 1.82
		*95% CI*	[0.65–1.16]	[0.55–1.06]	[0.32–0.98]	[0.69 –1.68]	[0.63–1.25]	[0.18–1.05]

Note: Δ = difference between t2 and t1 in *self-movement* and *object movement.*

**Table 4:** Analysis of Pearson’s correlations between *self-movement* and *object movement* and sex, age, BMI.

	1	2	3	4	5	6	7
(1) Sex	1						
(2) Age	–0.20 **	1					
(3) BMI	0.04	0.14	1				
(4) Object movement t1	−0.42 **	0.23 **	−0.10	1			
(5) Self-movement t1	0.22 **	0.05	–0.014	0.24 **	1		
(6) Object movement t2	−0.37 **	0.17 *	−0.08	0.69 **	0.29 **	1	
(7) Self-movement t2	0.12	0.03	−0.25 **	0.27 **	0.60 **	0.45 **	1

** *p* < 0.01, * *p* < 0.05.
